# ${\text{COSNet}}_i$: ComplexOme-Structural Network Interpreter used to study spatial enrichment in metazoan ribosomes

**DOI:** 10.1186/s12859-021-04510-z

**Published:** 2021-12-20

**Authors:** Federico Martinez-Seidel, Yin-Chen Hsieh, Dirk Walther, Joachim Kopka, Alexandre Augusto Pereira Firmino

**Affiliations:** 1 Willmitzer Department, Max-Planck-Institute of Molecular Plant Physiology, 14476 Potsdam-Golm, Germany; 2 School of BioSciences, University of Melbourne, Parkville, VC 3010 Australia; 3 Institute for Arctic and Marine Biology, UiT Arctic University of Norway, 9037 Tromsø, Norway

**Keywords:** Structural systems biology, Ribosome structure, Omics integration, Specialized ribosomes, Ribosomal protein substoichiometry

## Abstract

**Background:**

Upon environmental stimuli, ribosomes are surmised to undergo compositional rearrangements due to abundance changes among proteins assembled into the complex, leading to modulated structural and functional characteristics. Here, we present the ComplexOme-Structural Network Interpreter (${\text{COSNet}}_i$), a computational method to allow testing whether ribosomal proteins (rProteins) that exhibit abundance changes under specific conditions are spatially confined to particular regions within the large ribosomal complex.

**Results:**

${\text{COSNet}}_i$ translates experimentally determined structures into graphs, with nodes representing proteins and edges the spatial proximity between them. In its first implementation, ${\text{COSNet}}_i$ considers rProteins and ignores rRNA and other objects. Spatial regions are defined using a random walk with restart methodology, followed by a procedure to obtain a minimum set of regions that cover all proteins in the complex. Structural coherence is achieved by applying weights to the edges reflecting the physical proximity between purportedly contacting proteins. The weighting probabilistically guides the random-walk path trajectory. Parameter tuning during region selection provides the option to tailor the method to specific biological questions by yielding regions of different sizes with minimum overlaps. In addition, other graph community detection algorithms may be used for the ${\text{COSNet}}_i$ workflow, considering that they yield different sized, non-overlapping regions. All tested algorithms result in the same node kernels under equivalent regions. Based on the defined regions, available abundance change information of proteins is mapped onto the graph and subsequently tested for enrichment in any of the defined spatial regions. We applied ${\text{COSNet}}_i$ to the cytosolic ribosome structures of *Saccharomyces cerevisiae*, *Oryctolagus cuniculus*, and *Triticum aestivum* using datasets with available quantitative protein abundance change information. We found that in yeast, substoichiometric rProteins depleted from translating polysomes are significantly constrained to a ribosomal region close to the tRNA entry and exit sites.

**Conclusions:**

${\text{COSNet}}_i$ offers a computational method to partition multi-protein complexes into structural regions and a statistical approach to test for spatial enrichments of any given subsets of proteins. ${\text{COSNet}}_i$ is applicable to any multi-protein complex given appropriate structural and abundance-change data. ${\text{COSNet}}_i$ is publicly available as a GitHub repository https://github.com/MSeidelFed/COSNet_i and can be installed using the python installer pip.

**Supplementary Information:**

The online version contains supplementary material available at 10.1186/s12859-021-04510-z.

## Background

The function of cytosolic ribosomes is optimized to produce more ribosomes [1] through the translation of mRNAs. Translation creates ribosomal proteins (rProteins) that are used to produce functional ribosomes according to cellular needs. Moreover, translation builds the cellular machinery that initiates rRNA transcription and ribosome biogenesis, enabling processing of pre-ribosomes into translationally competent complexes [2–4]. Conceivably, ribosomes exist in various alternative forms, which vary structurally, and are functionally divergent, specialized complexes that meet translational requirements according to developmental or environmental cues [5–8]. Evidence for ribosome heterogeneity and specialization is rapidly growing across a wide variety of organisms [7, 9, 10].

Cytosolic ribosomes have a universal core that remained largely unchanged across evolutionary scales [11]. Compared to archaeal and bacterial ribosomes, metazoan cytosolic ribosomes logarithmically accumulated RNA expansion segments (ES) since approximately two billion years [11, 12]. Metazoan rProteins increased in number, duplicated, diverged, and acquired novel properties [13–17] which, when added to the accumulation of ES, implies extra potential to neo- and subfunctionalize. The ribosome considered as an entity is subject to selection and can be functionally specialized via heterogeneity of ES, rRNA modifications, substoichiometry of rProteins, i.e., the deviation from a canonical ribosomal proteome composition, the use of diverse rProtein paralogs or post-translational modification of rProteins and rRNAs [18, 19]. An important source of heterogeneity is rProteins substoichiometry, which can affect groups of rProteins [3]. In yeast, mutants deficient in individual rProteins can be defective in specific rRNA processing steps and consequently affect the assembly of multiple rProteins. Such defects are spatially constrained within the ribosome according to the sequence of ribosome assembly and thus depend on the overall location of the defective rProteins. Similarly, we expect that triggered structural heterogeneity may influence the assembly of specific rProteins, paralogs or post-translationally modified rProteins. Thereby, variants of ribosome complexes may arise with spatially constrained structural heterogeneity that extends across multiple adjacent rProteins. We hypothesize that such concerted structural heterogeneity may be at the core of ribosome specialization and influence the mRNA preference of mature ribosome complexes.

Available ribosome structures make it possible to test for spatial rearrangement in ribosomal complexes as a mode of functional specialization in response to specific cues. Such a test offers the possibility of integrating atomic structures and omics measurements of constituent ribosomal components. Integration of cryogenic or crystallographic atomic structures and omics data on abundances of structural components are part of the research field of structural systems biology [20] and begin with constructing a coarse-grained simplified representation of the structure, often represented as a graph. Using graphs, one can assign and compare node and edge-properties in order to answer biological questions at a single protein level [21]. Similarly, at the multi-protein level, structural models of protein complexes can preserve protein-protein interactions as edges connecting single protein components as nodes. Such networks enable topological analysis and comparison of node- and edge-properties. More detailed information on spatial relationships between proteins within a complex can be integrated by weighing the edges, where the edge weight describes specific properties of the interactions [22]. The edge weights can encode diverse properties, ranging from physical proximity to experimental evidence of said interaction. Using this approach, highly complex structures can be simplified to a network graph that represents essential structural information within orthologous protein complexes, such as the diverse variants of cytosolic ribosomes.

Cytosolic ribosomes readily lend themselves to a graph-based representation. These complexes are mixed ribonucleoprotein entities that consist of two subunits, namely the large 60S (LSU) and the small 40S (SSU) that combine to form a functionally mature 80S ribosome complex. Both subunits contain distinct ribosomal RNAs (rRNAs) as scaffolds for the binding of a multitude of rProteins [23]. If rRNAs, mRNA and tRNAs are excluded from the structural models, the outcome is an interconnected spatial array of rProteins that constitutes what we may call the structural ribosomal proteome (rProteome). A graph interpretation of the rProteome generates a specific topology that is the product of protein-protein interconnectivity and RNA mediated structural interactions generating community gaps within the network. Proteins within this network comprise sub-structures of physically adjacent entities. Thus, graph properties such as modularity [24], i.e., a measure of the division of a network into modules or communities, could be exploited to yield approximate rProtein communities. Likewise, coherent rProtein subsets can be sampled from these weighted rProteome networks. Random walks through weighted graphs are a well-documented procedure [25] capable of identifying communities within convoluted networks [26, 27] and correlations to hidden molecular functions. Community detection approaches enabled elucidating organizing principles of enzyme physical interaction networks and their relation to metabolic status [28]. Similarly, rProtein physical interaction networks provide the basis to define structurally coherent rProteome subsets that can be used to answer specific functional and biological questions. Going back to ribosome biogenesis, we may ask whether upon external cues, adjacent rProteins comprise significantly modulated sets of proteins.

Once coherent rProteome subsets are defined, these can be analyzed to identify localized changes based on systems biology data. Transcriptomic measurements of rProtein gene expression changes can be considered as a first level of information integration, supporting prediction and hypothesis generation. On the other hand, measurements of rProteome composition can verify assumptions of localized changes within the ribosome complex. The spatial enrichment analyses proposed in this manuscript contribute to the prediction and verification of ribosome heterogeneity, e.g., substoichiometry of ribosome complexes or changes in rProtein paralog composition, and more importantly, add the aspect of concerted ribosome heterogeneity affecting sets of co-localized rProteins. Concerted heterogeneity can be expected, as ribosome biogenesis is a highly regulated sequential process that is far from random. Alternatively, post-assembly changes are conceivable but restricted to surface accessible rProteins. Modulation of spatially-linked groups of rProteins rather than heterogeneity of single rProteins may be the basis of ribosome specialization and confer ribosome complexes the ability to influence the translational status of transcripts, favoring those that require active translation upon environmental or developmental cues, a concept known as the “ribosomal code” [29].

In the current study, we present a workflow enabled by the ComplexOme-Structural Network Interpreter (${\text{COSNet}}_i$) python module that decomposes cryogenic or crystallographic atomic structures of multi-protein complexes into subsets of physically adjacent proteins and subsequently tests them for enrichment of concerted changes relative to other parts of the complex. Thereby we integrate structural information with readily available omics-measurements from systems analyses. To achieve this integration, we subset protein interaction networks of multi-protein complexes derived from elucidated structures using a random-walk sampling with restart. Structural coherence and region consensus is achieved by iterating the sampling procedure through a translated graph weighted by protein physical proximity as a proxy of traversal probability. We test the performance of ${\text{COSNet}}_i$ by comparing regional coherence with several graph community detection algorithms. We highlight as a novelty that ${\text{COSNet}}_i$, unlike the tested algorithms, allows users to customize coherent regions for specific biological questions. Consequently, we describe a procedure to optimize parameters of our sampling and evaluation method using as case studies the cytosolic ribosome complexes of various metazoans. More specifically, we compare the relatively simple yeast ribosome to the more complex mammalian and plant counterparts and integrate available systems data of each species. To gather information on a previously unanswered biological question, we explore concerted localized rProtein heterogeneity that suggests ribosome specialization. We specifically ask whether changing physiological conditions affect rProtein heterogeneity in a way that is constrained to specific spatial regions of metazoan ribosomes.

## Implementation

**Fig. 1:**
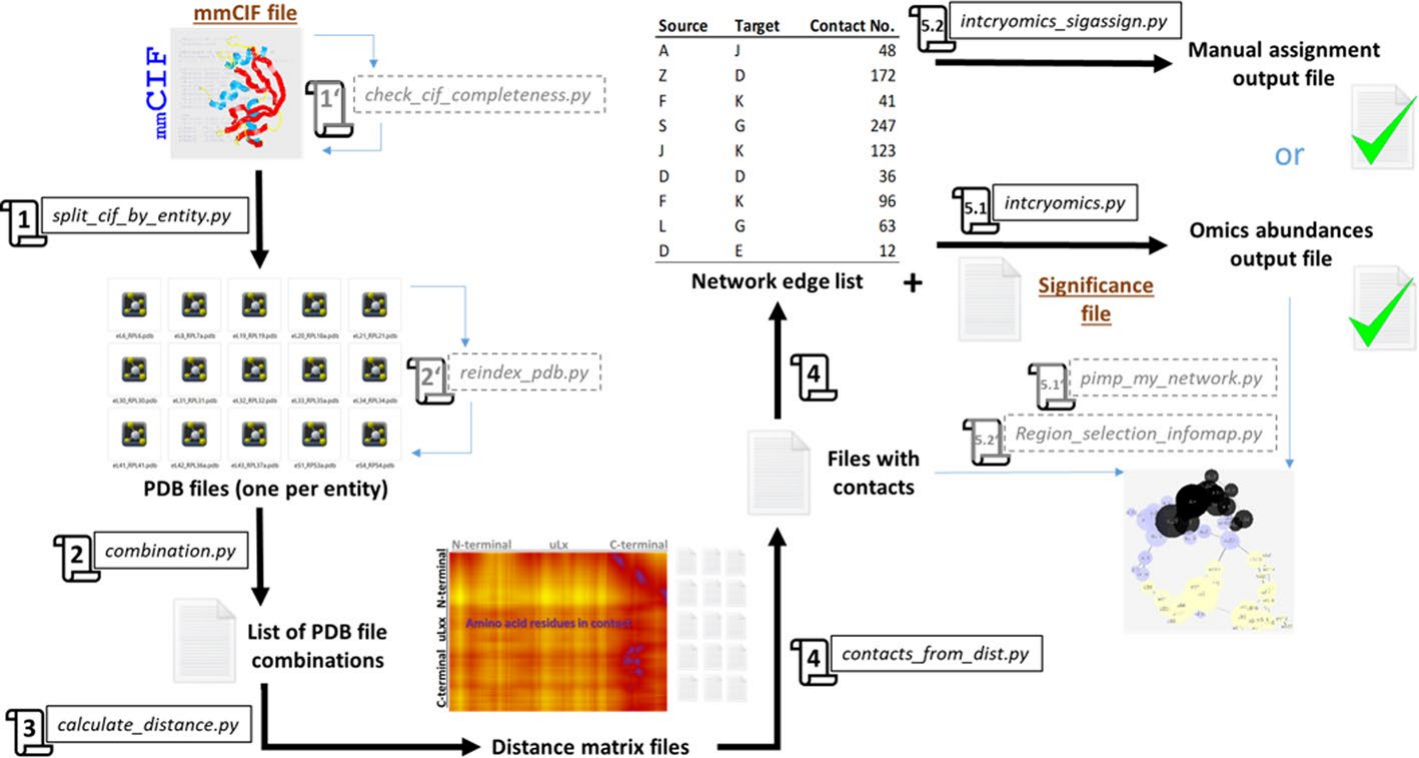
COSNet$_i$ step-by-step detailed workflow. Also related to Additional file 1 where the two detailed examples from this manuscript were optimized and developed. ${\text{COSNet}}_i$ is divided in five steps that must be completed plus accessory functions that allow users to perform quality control checks or produce alternative outputs along the way. The input data is an mmCIF file. Step 1 extracts all the protein entities from the input file as PDBs using *split_cif_by_entity.py*. Additionally, Step 1’ allows checking the percentage of coverage of each modelled protein sequence as compared to its FASTA sequence using *check_cif_completeness.py*. Step 2 prepares the PDB files by building a list of combined names for each protein pair using *combination.py*. In parallel the workflow offers as Step 2’ the opportunity to reindex the residues column inside PDBs in case there are disruptions in the structures that would lead to holes by using *reindex_pdb.py* or its batch counterpart *batch_reindex_pdb.py*. Step 3 takes the list of PDB combinations and fits distance matrices across each file pair using *calculate_distance.py* or its batch counterpart *batch_calc_dist.py*. Step 4 uses the distance matrices to build a list of contacts and a graph through the use of *contacts_from_dist.py*. Finally, Step 5.1 integrates the Omics abundances into the graph analyses through *intcryomics.py*. Alternatively, if there is not a binary Omics file users may rely on Step 5.2 *intcryomics_sigassign.py* to manually select the protein entities that feature significant changes. Step 5.1’ returns customized graph files that can be used to highlight specific regions in the networks using *pimp_my_network.py* while Step 5.2’ allows users to investigate structural coherence in the regions selected through the existing graph community detection algorithm Infomap using *Region_selection_infomap.py*. mmCIF icon was taken from IUCr

${\text{COSNet}}_i$ is a python module organized based on a collection of scripts that allow any user to select coherent spatial neighborhoods of protein entities from a multi-protein complex in order to test whether these communities characterize a region within the complex that becomes significantly enriched upon any experimental procedure. The complete workflow is detailed in a step-by-step manner in Fig. 1 and Additional file 1.

### Structural data preprocessing

RCSB PDB entries 6SNT, 6GZ5, and 4V7E were retrieved as PDBx/mmCIF files and the following pre-processing steps were implemented to ensure their usability for this study. Nonstandard amino acids labeled as “hetero atoms” (HETATMs) and duplicate atoms were removed from all proteins. rRNA, ions, tRNA, and mRNA components of the original structure were ignored. The percentage of missing residues per ribosomal protein (rProtein) was noted (see Structure Quality Requirements section in the Discussion). Each rProtein sequence was verified as correctly labeled via BLAST [30] against the protein entry originally modelled into the Cryo-EM densities. Proteins were renamed according to the new rProtein naming scheme [31].

### Proximity network building

Translating ribosome atomic structures to rProtein proximity networks allowed characterization of the overall topology and protein relative positions. The structural interactions of rRNA and rProteins were not considered because the rRNA structures were ignored when building the network. Thus, the concept of proximity in the current study does not imply direct physical interaction between rProteins, rather a high potential for interaction due to physical proximity. The resulting network is an undirected graph, with nodes representing proteins and weighted edges between two proteins sharing at least one spatial contact at a given proximity threshold. To calculate contacts between proteins, all amino acid residues belonging to both proteins were represented in the three-dimensional space of coordinates of the given model by their geometric center of mass (i.e., coarse-grained to a single point). The Euclidean distances between each pair of amino acids from paired rProteins were calculated. The choice to coarse-grain at amino acid residue level enabled detection of potential interactions of extended non-globular proteins that branch out far across the ribosome, such as universal large ribosomal protein 4 (uL4) or eukaryotic large ribosomal protein 19 (eL19). Edges were accepted at different distance thresholds (e.g., $d_t$ = 5, 8, 12, or 20 Ångströms [Å]). Thereby, we generated several proximity networks varying around the common consensus of 8 Å for residues to be considered in contact, according to the 8th Critical Assessment of Protein Structure Prediction experiment (CASP8) [32]. Weighting of edges was performed according to the proportion of inter-amino acid residue contacts found between two proteins as compared to all the inter-amino acid residue contacts from the source node-protein. Networks were visualized with the R package *igraph* [33] and Cytoscape software [34].

### Structural region definition

**Fig. 2:**
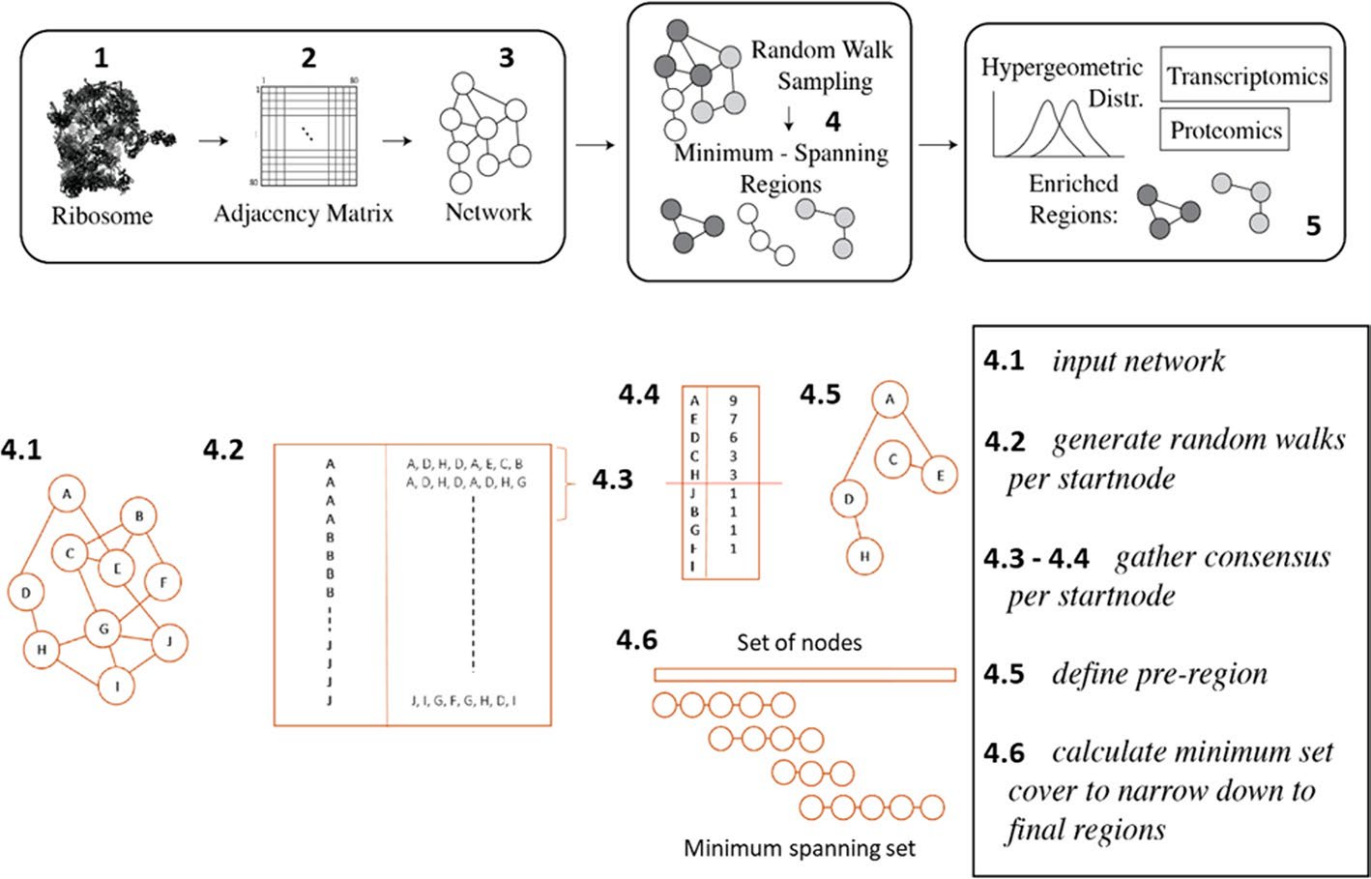
COSNet$_i$ workflow emphasizing the novelties within our consensus random walk sampling procedure. Illustration of structural sampling and testing methodology used in ${\text{COSNet}}_i$ to test whether proteome heterogeneity is spatially confined in multi-protein complexes. The upper panel depicts the workflow, divided into three parts: proximity network building from structural data, consensus random walk sampling based on the input network, and statistical testing of the defined regions. The lower panel shows in-depth the novelties within the consensus random walk sampling procedure

Splitting ribosomes into separate structurally coherent regions allowed for targeted statistical testing of protein features within regions (Fig. 2—upper panel) and ensured that any regions of interest could be further validated by known biological domains. The main priority was avoiding pre-knowledge biases while selecting node associations. To achieve this, randomness was introduced when sampling nodes. In detail, a consensus random walk sampling procedure with restart methodology was implemented. The procedure (Fig. 2—lower panel) involved: (4.1) a proximity network was taken as input, and a walk length and iteration number were defined. The walk does not reverse and is completely memoryless. The walk length represented the number of steps a random walk takes before terminating, and the iteration number was the number of random walk restarts from a particular starting node. Edges between protein nodes were weighted based on the number of amino acid residues in contact normalized by the number of amino acid residues of the source node and transformed into a transit probability. Given two protein nodes, *x* and *y*, the probability of walking from node *x* to *y* is computed as $P_{x,y} = w_{x,y} / w_{x}$, where $w_{x}$ is the sum of all weights of all outgoing edges of node *x*. Thus, the probability corresponds to how many contacts there are between node *x* and *y*, relative to all other nodes connected to *x*. The random walk is no longer purely ’random’ in the strict sense, but has a higher probability to walk along an edge with a higher weight. (4.2) A collection of all walks for every start node for all nodes in the network was compiled. Exemplary sample walks for start nodes A and J were selected for illustration purposes (Fig. 2—lower panel step 4.2). (4.3) For all sets of walks that share the same start node, (4.4) a count-based summary of node visits was calculated, where every instance of a visit to a node, even those within the same walk, was tallied. In our example (Fig. 2—lower panel step 4.4), walks with start node A often visited nodes E and D, followed by visits to nodes C and H. (4.5) Pre-regions were defined for all start nodes, consisting of nodes that were visited with a frequency of at least half of the iteration number. Using a count-based consensus ensured that nodes, which were relatively far away from the start node and were visited by chance, were excluded from the pre-regions. As an example, the pre-region for start node A is A, E, D, C, H (Fig. 2—lower panel step 4.5). Steps (2-5) were carried out to ensure that the pre-regions were not biased towards a single walk from a certain start node and also that each node in the network served as start node. Thereby, all nodes were visited at least once. At this point, the number of pre-regions equaled the number of nodes in the network since each of the nodes served as starting point. The level of node overlap among the pre-regions varied, where two pre-regions with different start nodes could in one extreme case be fully distinct from one another or in the other extreme be identical. (4.6) Final regions were aggregated from the pre-regions by calculating the minimum set cover that spanned the entire universe of protein nodes. This procedure gave the minimum number of final regions that spanned the entire node space, and returned a small set of regions with minimized redundance. Finding the minimum set cover gave preference to large and more complete regions that mapped to the entire node space, as opposed to a large number of small regions.

### Testing of enriched relative changes within regions

The statistical testing procedure used the set of all known rProtein paralogs and aimed to discover whether there is an association between protein nodes being part of a structural region, and having changed in relative abundance (CRA) in response to experimental conditions. CRA was defined as differential stoichiometry between ribosomal complexes as determined by proteomics data. CRA was defined as a binary data-type, where a code of “1” indicates abundance changes and a code of “0” indicates otherwise. The testing scheme assumes a background hypergeometric distribution, and is thus equivalent to the Fisher’s exact test, with baseline probability of enrichment equal to the total fraction of paralogs with CRA compared to all rProtein paralogs. The null hypothesis here states that there is no relationship between being part of a particular structural region and having the CRA property. In other words, the null hypothesis assumes that proteins exhibiting the CRA property are distributed randomly throughout the complex. For statistical testing, the SciPy implementation of the Fisher’s exact test was used [35]. Due to multiple testing, computed *p*-values generated by the Fisher’s exact test were adjusted via Bonferroni correction [36].

### Test case datasets

Three ribosome structures were used in order to optimize the parameters of our methodology. All datasets corresponded to metazoans ribosomes with varying complexities. More specifically, the ribosome structures of *Saccharomyces cerevisiae*—2.80 Å (https://www.rcsb.org/structure/6SNT), *Oryctolagus cuniculus*—3.50 Å (https://www.rcsb.org/structure/6GZ5), and *Triticum aestivum*—5.50 Å (https://www.rcsb.org/structure/4V7E) were used. The datasets varied in structural resolution, which allowed us to determine whether a relatively low resolution would preclude the use of our method (see Structure Quality Requirements section in the Discussion). In agreement with these considerations only two exemplary structures were tested for spatial rearrangements of the riboproteome. The third (i.e., the only available plant cytosolic ribosome structure) one should be used carefully considering the parameters provided in ${\text{COSNet}}_i$. We selected proteomics datasets that indicated substoichiometry of rProteins in mammalian cell cultures and yeast. The following selected datasets evaluated rProtein substoichiometry between pools of free non-translational subunits or monosomes and translationally competent polysomes:

**1.** Mammalian [37] taken from Shi et al. (2017). **Species**: Mus musculus. **Cell line**: Low-passage E14 mouse embryonic stem cells (mESCs). **Riboproteome**: Additional file 2 from Shi et al. (2017). rProteins that were significantly substoichiometric, i.e., $P < 0.05$ were set to “1”, similarly proteins that did not have a statistical change with $P > 0.05$ were set to”0”. Ribosomal protein coding genes and paralogs have been compiled from Supplementary Table 1 from Perry (2005) [38] by translating the nomenclature into the common new rProtein family names [31]. If the sequence of significantly changed paralog rProteins within one family was identical, all paralogs were set to one.

**2.** Yeast [39] taken from Slavov et al., (2015). **Species**: Saccharomyces cerevisiae. **Cell line**: “prototrophic diploid strain (DBY12007) with an S288c background and wild-type HAP1 alleles (Slavov and Botstein, 2011)”. **Riboproteome**: Additional file 4, mmc5. Additional file 4 treated paralog ambiguities as a united rProtein family response. Thus, the top substoichiometric rProteins, including all paralogs per family, with a larger than 0.5 absolute $log_2$-fold change among translating polysomes loaded with different amounts of monosomes were set to “1”, the rest of the proteins were set to “0”. The complexes were isolated from glucose-fed yeast, growing at stationary rate, and recovered from ribosomal fractions corresponding to four loaded 80S-ribosomes per mRNA. rProtein coding genes and paralogs have been compiled from the Saccharomyces Genome Database (SGD) by translating the nomenclature into the common new rProtein family names [31].

In both cases, the entire set of rProteins was considered as all the paralogs from the proteins that were available in the structural files. Therefore, to prevent false significances, the annotated peptides were verified against the FASTA sequences of paralogs within rProtein families to make sure that they were not redundant. In case of redundancy, both paralogs were considered to have contributed to the sequenced peptide identified protein and thus were set to “1” if significant.

## Results

The ${\text{COSNet}}_i$ workflow, outlined in Figs. 1 and 2, can be generalized to accommodate any multi-protein complex as long as paired orthologous structures and differential omics abundances are available. Numerical parameters such as the structural proximity threshold ($d_t$) and module-sampling related walking length need to be tuned based on prior analyses of size and resolution-quality of the studied complex. In the following sections, we use the cytosolic ribosome as a test case to exemplify the fine-tuning of those parameters. As is explained in our introduction, we aimed the method towards analysis of the rProteome, i.e., the compendium of structural rProtein components. Consequently, parameter optimization coped with the intrinsic proteome diversity that our test cases, the metazoan ribosomes, have. We analyzed structures from less complex riboproteomes that contain only one to two paralogs per rProtein, i.e., the yeast and mammalian riboproteomes. As more complex cases, we selected the highly complex plant riboproteome, which potentially harbors combinations of two to seven paralogs per rProtein family in the dicot model plant Arabidopsis [40] or two to three per rProtein family in the monocot example, rice [41]. The canonical structures of ribosome complexes accommodate single copies of each rProtein. Therefore, we designed the procedure to perform regardless of the number of paralogs per rProtein family and organism. We chose to always test the whole set of annotated rProtein paralogs per genome, thereby using the near comprehensive information from omics studies.

### Translating structures into graphs

**Fig. 3:**
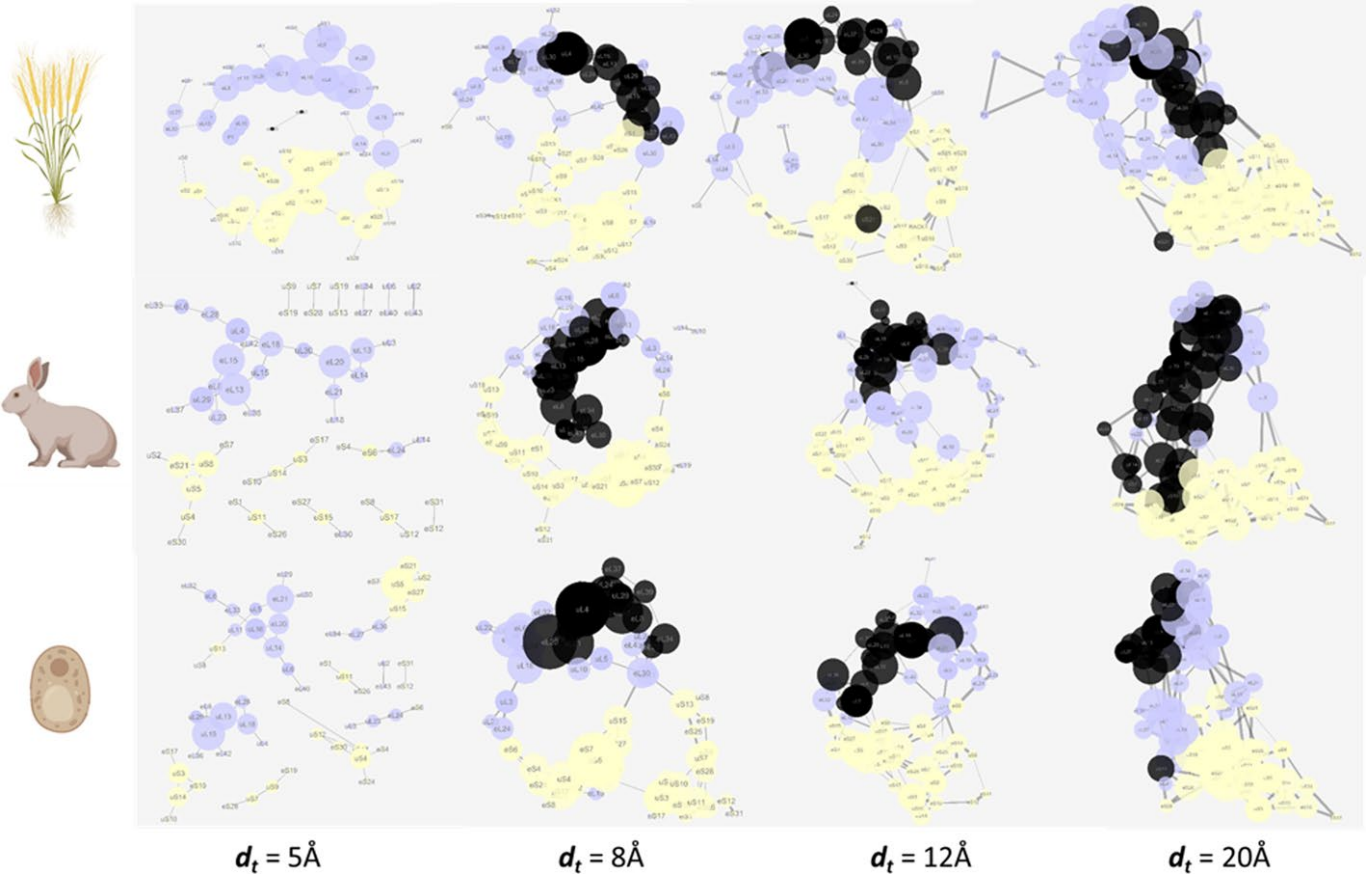
Ribosomal protein networks at different distance thresholds (d_t_) between amino acid residues in contact. Highlights of the polypeptide exit tunnel yielded region are outlined in black as a measure of structural and biological accurateness of the obtained networks. The networks were built using the ${\text{COSNet}}_i$ workflow (https://github.com/MSeidelFed/COSNet_i) with default values from PDBx/mmCIF entries 6SNT, 6GZ5, and 4V7E corresponding to *Saccharomyces cerevisiae* (bottom panel), *Oryctolagus cuniculus* (middle panel) and *Triticum aestivum* (upper panel) ribosome structures. The networks were analyzed as undirected graphs in Cytoscape [34], a larger node size indicates larger degree, the thickness of edges is defined as a transit probability between nodes calculated based on the number of contacts between each protein pair and the network layout is edge-weighted spring-embedded to simulate a real structurally connected network with forces acting upon it. The 60S subunit nodes have been highlighted in light blue/black, and the 40S subunit nodes in light yellow, nodes that belong to the PET region of the 60S LSU (i.e., region containing eL39 rProtein family) have been highlighted in black. Note that as $\hbox {d}_t$ gets larger outlier proteins get into the defined regions while when $\hbox {d}_t$ is lower rProteins are not fully interconnected and many nodes are missing. Species icons were exported from BioRender (https://biorender.com/) under a paid license. The network interactions and weights have been compiled in Additional file 2

The first critical parameter to obtain a weighted graph from an atomic structure is the definition of a distance threshold that determines the adjacency matrices between protein nodes, and ultimately influences the resulting network of nodes and edges. According to CASP8, the consensus distance for a residue-residue contact within a protein structure is 8 Å [32]. More specifically, residues in contact have their C$\beta$ atoms ($\beta$-carbon or C$\beta$, or C$\alpha$ for glycine) within a distance of 8 Å. Nevertheless, as many rProtein interactions are mediated by rRNA molecules, we tested whether the 8 Å threshold correctly reflects the structure of the ribosome in the obtained protein network. The aim of a network representation is to simplify the three-dimensional atomic models, while retaining structural and biological accuracy. Thus, the proximity network topology must reveal known ribosome structures as an internal means of validation. To investigate the biological accuracy of our networks, the clustering behavior within both ribosomal subunits, i.e. the LSU and SSU, was determined at different distance thresholds (Fig. 3). Our network layouts treated the edges between nodes as elastic springs. The springs organized themselves according to a force function influenced by the weight of each edge. The function minimized the sum of forces in the network, i.e. Edge-weighted Spring-Embedded algorithm in Cytoscape [34]. This layout algorithm treats a network as an interconnected structure of actual physical interactions. The rearranged network allowed us to describe topological features of the complexes that support biological knowledge (Fig. 3).

The topology of the proximity networks at varying distance thresholds ($d_t$ = 5, 8, 12 and 20 Å see Additional file 2) outlined structural features of the ribosome. A consensus random sampling was done for one exemplary variable region, i.e., the polypeptide exit tunnel (PET), characterized by at least the eL39 and eL37 protein families (PET in Fig. 3). It became evident that varying thresholds affected region coverage. Increasing the distance threshold resulted in increasing variability of defined regions. In other words, a higher threshold included “outlier” proteins, which were not physically close to the canonical rProtein cluster of the region. By contrast, if the threshold was too small, the network contained separate islands with some expected nodes omitted from the network. Hence, the outcome was a low connectivity among rProteins. An ideal distance threshold should produce a network, in which all the expected nodes or constituent proteins of the structure link by at least one edge. The possible optimized outcomes are a compromise between connectivity and coverage (Table 1).

**Table 1:** Compromise between connectivity and coverage among networks fitted at varying distance (Å) thresholds

Species	Nodes	Edges	Threshold
*Triticum aestivum*	*68*	**73**	5 Å
	*75*	**131**	8 Å
	*78*	**172**	12 Å
	*79*	**203**	15 Å
	*80*	**238**	18 Å
	*80*	**263**	20 Å
*Oryctolagus cuniculus*	*59*	**49**	5 Å
	*71*	**123**	8 Å
	*75*	**164**	12 Å
	*77*	**200**	15 Å
	*77*	**232**	18 Å
	*77*	**263**	20 Å
*Saccharomyces cerevisiae*	*60*	**57**	5 Å
	*67*	**115**	8 Å
	*68*	**146**	12 Å
	*71*	**180**	15 Å
	*71*	**207**	18 Å
	*71*	**227**	20 Å

*Italic* style represents the node number and **bold** the edge number

Considering the PET region, at $d_t$ = 5Å, the PET rProteins eL37 and eL39 were only visible in the wheat structure due to a single mutual link, while eL39 was not at all included in the yeast and rabbit networks (Fig. 2). Similarly, more than 15% of the rProteins were omitted from the three networks at $d_t$ = 5Å (Table 1). At the other extreme, with $d_t$ = 20Å, the entire network is highly inter-connected. An indication of over-represented connectivity is the transition to an exponential rate at which the number of edges increases relative to the nodes with increasing d_t_ (Table 1). Returning to our example (Fig. 3), the PET is a densely packed region that increased in size with increasing $d_t$ beyond the canonical PET definition and contained a large proportion of LSU proteins in the rabbit and wheat networks. Problems became apparent, too, with across structure interactions. For instance, rProteins found at opposite parts of the ribosome were included into the same region in wheat already at $d_t$ = 12Å, while this happened in the yeast case only at $d_t$ = 20Å. Inversely at $d_t$ = 8Å, the plant P-stalk proteins became disconnected from the network. Disconnection creates a bias in as much as at every sampling step the P-Stalk will be an isolated region. Upon inspection of the wheat structure, we realized that this problem arises due to partially incomplete rProtein sequence coverage (see Structure Quality Requirements section in the Discussion). For this structural quality reason, we omitted the plant structure from the following analyses. The rabbit and yeast networks were 95% connected at $d_t$ = 12Å, without isolated sub-regions, while “outlier” proteins were still absent. We therefore identified $d_t$ = 12Å as the ideal distance based on which to define regions in the yeast and rabbit structures. Using the same concepts, in the wheat structure a $d_t$ = 8Å would be the preferred threshold, were we to proceed with this analysis as concurrently done [42]. The chosen distance thresholds covered at least 95% of the nodes in all three cases.

### Defining spatial regions

**Fig. 4:**
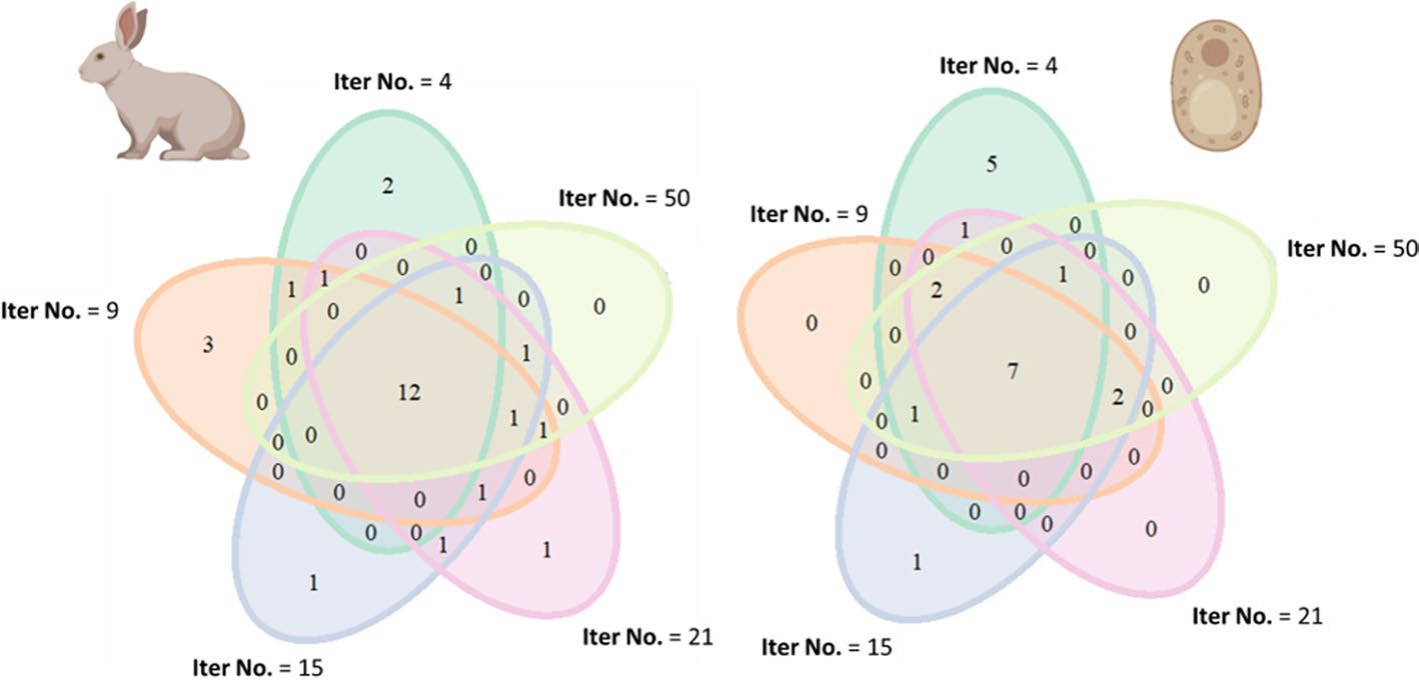
Overlap between Obtained PET Regions of Yeast and Rabbit at Varying Iteration Number for Consensus. The *intcryomics.py* function was run using a default walking length of $\sim \frac{1}{4}$ of the network nodes (i.e., walking length of $\sim 20$ nodes) and 4, 9, 15, 21, or 50 iterations. The resulting regions that contained the PET signature rProteins, i.e., eL39 and eL37, were concatenated for a single run, and intersected with the resulting PET regions from other runs. The results from the intersection were visualized using Venn diagrams with the VennDiagram [74] package in R software [75]

Once a network is compiled, walking across the network requires a predefined number of random steps and definition of a starting node. The direction of each step of the walk is influenced by the edges weights and the node interconnectivity. The clustering coefficient of the starting node is a determinant of the defined regions. The clustering coefficient is a measure of connectivity among neighbors of the starting node. A high clustering coefficient of the starting node means that a random walk will stay in the vicinity or may even return to the starting node. By contrast, if the starting node has a low clustering coefficient and high betweenness centrality, the walk will likely lead to one of the parts of the network that the starting node connects. The measure of betweenness centrality refers to node importance in a network. A node that often acts as a bridge within shortest paths across the network has high betweenness centrality and connects largely separate modules within a network. In this sense, our methodology defines densely packed regions or modules of nodes in the weighted graph. Hubs that connect modules are attached to the closest group of highly interconnected neighbors. In order to avoid bias of a single walk trajectory, we iterate the random walk following a restart methodology from each node by a predefined number of times. The iteration number has high impact in the reproducibility of obtained regions and increasing it achieves region consensus, e.g., our exemplary analysis of PET variability (Fig. 4). After each walk, we gather a consensus of the most visited nodes from each start node and form pre-regions. Initially, the number of pre-regions equals the number of nodes. The following steps reduce the number of regions to a minimum set that covers the whole network with minimum overlap between regions.

In general, a core PET region occurred in every consensus, with seven nodes in the yeast and twelve nodes in rabbit network. When the iteration number was small, outlier proteins that did not belong to the canonical regions tended to be part of them after the network sampling procedure. For example, five specific nodes for yeast and rabbit are only part of the consensus walk when the iteration number is smaller than 10 in Fig. 4. This exemplifies the necessity of iterating the consensus walk to increase the reproducibility in region picking. As the iteration number increased, fewer outlier proteins, product of biased consensus walks, were found. Proteins from a consensus replaced outlier proteins from low iterated walks at higher iteration numbers. For instance, there were three and two proteins shared by Iteration No. 21 and Iteration No. 50 in the rabbit and yeast PET region, respectively (Fig. 4).

In addition to the iteration number, the walking length parameter (i.e., number of steps in a walk) influenced the size and number of regions identified as the minimum spanning set covering all nodes. Here, we exemplify how the resulting ribosomal regions varied due to taking different proportions of the total node set as walking length (Table 2 and Additional file 3). Going beyond or below the proposed proportions may suit different computational needs and biological questions. In our case, we aimed at testing the relative proportion of significantly changed nodes in the resulting regions as compared to the whole ribosome. Therefore, regions with varying degrees of overlaps are acceptable. If partially overlapping regions have a different significance *p*-value, it means that not all structurally related proteins from a given region are changed, rather a specific combination of rProteins needs to be changed in order to call a region significantly enriched.

**Table 2:** Tuning the walking length enables yielding region sizes needed for specific biological questions

Species	Node proportion/set	0.5	0.33	0.25	0.2	0.17	0.14	0.13	0.11	0.1
*Oryctolagus cuniculus*	77	39	26	19	15	13	11	10	9	8
Region number		8	11	12	14	15	19	17	21	21
Average region length		15	12	10	8	7	7	6	5	5
1st quartile		10	10	8	7	6	6	5	4	4
Median		17	12	9	8	7	7	7	5	5
3rd quartile		19	14	12	9	8	7	7	6	6
*Saccharomyces cerevisiae*	71	36	24	18	14	12	10	9	8	7
Region number		13	12	13	15	16	18	19	20	22
Average region length		17	13	11	9	8	7	6	6	5
1st quartile		15	11	10	8	6	6	5	4	5
Median		17	14	11	9	8	7	7	7	5
3rd quartile		18	15	13	10	8	8	7	7	6

Region size needs can vary with different biological questions. To cover variable regions sizes we introduced the walking length parameter, which, when increasing, progressively yields larger regions (Table 2 and Additional file 3). The defined regions could then be tested for enrichment based on abundance changes of their constituting proteins. In the ribosomal example, we optimized the region size to match the significant proportion of nodes. In other words, regions had to be large enough for a single node to be proportionally equivalent to the percentage of significant nodes in the network. For instance, if 20% of the nodes in the network were significantly changed, i.e., showed evidence of changed abundance, then the region must be at least five nodes in length in order to have one significant node meeting the background proportion. Smaller regions imply that a changed node may be interpreted as a local enrichment of changed abundance in the multi-protein complex. This becomes especially relevant when the percentage of significant nodes in the network is low. The proportion of significant nodes in our ribosome test cases varied (Additional file 4). Therefore, we tuned the average region sizes to contain at least the number of rProteins that would make a single unit equivalent to the baseline proportion of significances. This enabled us to test whether rProtein dependent ribosome specialization is locally enriched in ribosomal regions.

### Building a ribosomal protein network

**Fig. 5:**
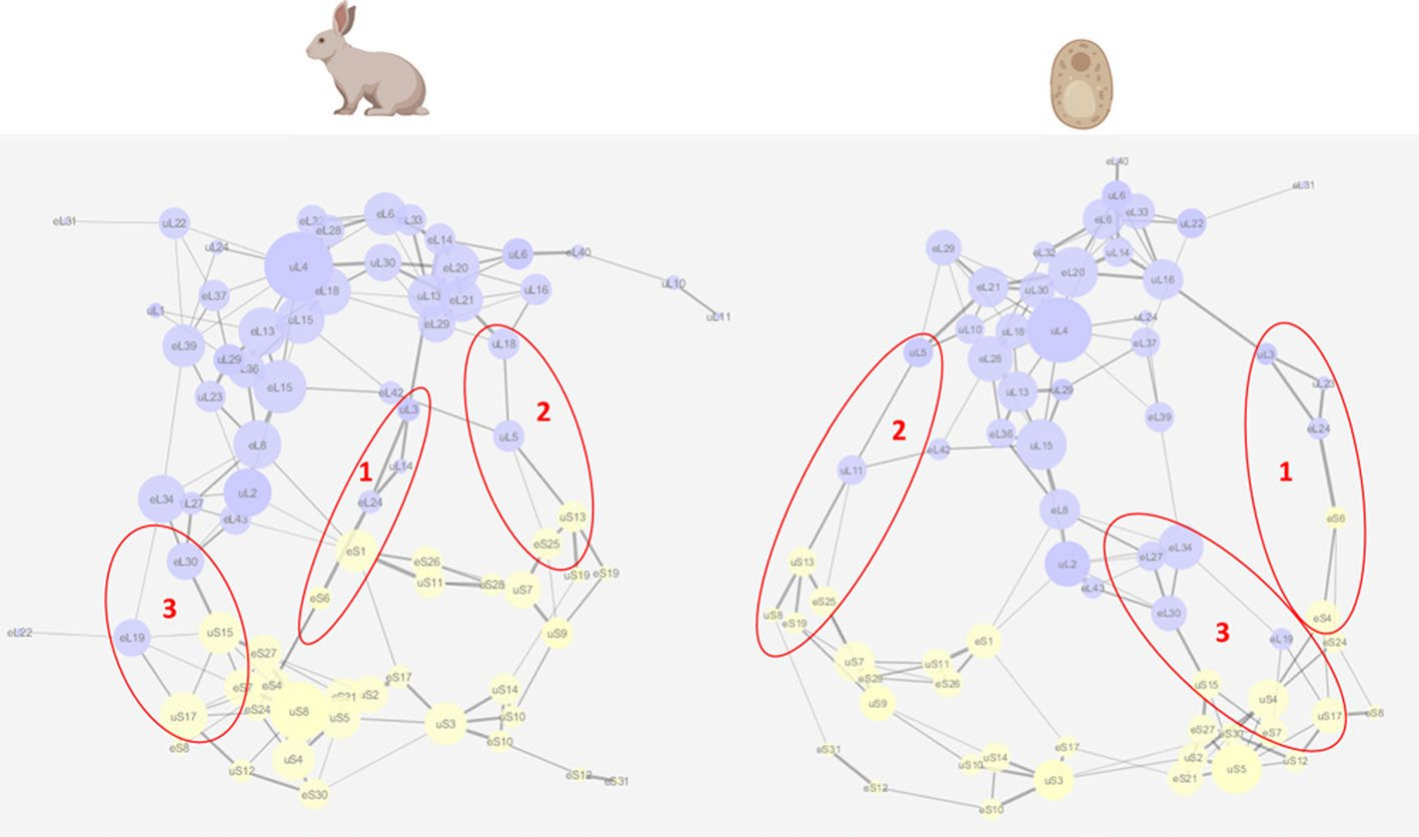
Optimized yeast and rabbit ribosomal protein networks. **a** *Saccharomyces cerevisiae* network, **b** *Oryctolagus cuniculus* network built at a contact threshold ($d_t$) of 12 Å between amino acid residues. The network layout is Edge-weighted Spring-Embedded. The weights of edges correspond to the number of contacts between two rProteins and in that sense are proportional to the transit probability defined as the main influence during ${\text{COSNet}}_i$ random walk. A larger node size corresponds to a larger node degree. Nodes belonging to the 60S large subunit (LSU) have been colored blue and nodes belonging to the 40S small subunit (SSU) have been colored yellow. Note that there are three conserved/sampled interface pathways between rProteins from the two subunits (Table 3). The network representations have been created in Cytoscape [34]

The consensus networks for our ribosomal test case were built with a threshold of 12 Å that allowed a coverage > 95% of the nodes corresponding to 75 and 68 nodes (proteins) in the rabbit and yeast networks, respectively. Secondly, we made sure that the region selection did not contain outlier proteins by iterating the region consensus 50 times. Finally, a random walk length equal to 0.13 times the node set was selected. This proportion achieved regions sizes that enabled us to match the baseline probability of significances for the prioritized test cases (see Testing the Spatial Constraints of Ribosome Specialization and Additional file 4) in both networks, yeast and rabbit. Subsequently, we compared the resulting optimized ribosomal networks (Fig. 5). We uncovered interconnected paths, highly or poorly interconnected rProtein neighborhoods, dense module and inter-module connective hubs, bridges between important structural features and biological details that where either conserved or different between the investigated organisms (Fig. 5).

**Table 3:** Matching of coherent regions with biologically known network topologies

Regions	rProteins	Region identifiers
Rabbit—60S rProteins		IntCryOmics_6gz5_d_t_12_IN50_WL10
Region 1	eL32 uL4 eL14 eL20 eL6 uL13 eL28 eL33	LSU-TopRegion
Region 2	eL13 eL15 eL36 uL15 uL29 uL23 eL8 uL2	LSU-Center.2
Region 10	eL13 eL31 uL22 eL39 eL37 uL29 uL4	LSU-PET.1
Region 11	uL11 eL40 uL10 uL6	LSU-P-Stalk
Region 12	eL18 uL4 eL39 uL24 eL28 uL30	LSU-PET.2
Region 17	eL20 eL21 eL29 uL30 uL18	LSU-Interface2Adjacent.1
Region 16	eL13 eL15 eL36 uL15 uL1 uL29 eL8	LSU-Center.1
Region 19	eL14 eL20 eL21 uL16 uL18	LSU-Interface2Adjacent.2
Subunit interface rProteins		
Region 5	eL13 eL15 uL5 eL42 eL21 uS13 uL18	Interface2
Region 6	uS12 eS8 eL19 uS15 uS17 uS8	Interface3.2
Region 8	eL27 eL30 eL43 uL2 eL34 uS15	Interface3.1
Region 9	uL3 eL24 eS6 uL14	Interface1
Region 18	uS12 eS8 eL19 eL22 uS17	Interface3.2
40S rProteins		
Region 3	eS24 eS4 eS30 uS2 eS21 uS4 uS5 uS8	SSU-Interface3Adjacent
Region 4	eS25 uS7 eS28 eS1 uS9 eS26 uS11	SSU-Interface1Adjacent
Region 7	uS10 eS10 uS14 uS3 eS17 uS2	SSU-CentralHub
Region 13	eS7 uS15 eS21 eS27 uS5 uS8	SSU-Center
Region 14	eS12 uS3 eS31 eS10	SSU-BottomTail
Region 15	uS7 uS19 eS19 uS13 uS9	SSU-Interface2Adjacent
Yeast—60S rProteins		IntCryOmics_6snt_d_t_12_IN50_WL9
Region 2	eL8 uL2 eL28 eL36 uL13 uL15 eL43	LSU-Interface3Adjacent.1
Region 3	eL31 uL22 eL6 eL20 eL33 uL14 uL16	LSU-TopRegion.1
Region 6	eL39 eL37 uL29 uL4 eL28 uL13 uL18	LSU-PET
Region 11	eL20 eL21 uL10 uL30 uL5	LSU-Interface2Adjacent.1
Region 12	eL6 eL20 eL40 uL14 uL16 uL6	LSU-TopRegion.1
Region 15	eL34 eL27 eL30 uL2 eL43	LSU-Interface3Adjacent.2
Region 16	eL20 eL21 uL30 eL29 uL5	LSU-Interface2Adjacent.2
Region 17	eL6 eL33 uL14 uL16 eL32	LSU-TopRegion.3
Region 18	uL24 uL30 uL4 eL28 uL13 uL18	LSU-Center
Subunit interface rProteins		
Region 1	eL19 uS12 eS7 uS17 eS8 uS5 uS15	Interface3.2
Region 4	eL21 uL11 uL5 uS13 uS7 eL42 eS25	Interface2
Region 5	eL24 eS6 uS2 uS4 eS24 eS4 eS30	Interface1
Region 8	eS7 eS21 eL27 eL30 eS27 uS5 uS15	Interface3.1
Region 13	eS6 uL23 uL3 eL24	Interface1
40S rProteins		
Region 7	uS10 eS10 uS14 uS3 eS17	SSU-CentralHub
Region 9	eS1 uS7 eS26 uS11 eS28	SSU-Interface3Adjacent
Region 10	uS8 eS19 uS13 uS7 uS9 eS25	SSU-Interface2Adjacent
Region 14	eS10 eS12 uS3 eS31	SSU-BottomTail

Both networks separated into 60S LSU and 40S SSU. Subunits were connected via nodes with a high betweenness centrality and a low clustering coefficient. There were four interface paths connecting both subunits. Path number one conserved rProtein families uL3, eL24 and eS6 as the main transit nodes. In yeast, there was an additional edge connection between eL24 and uL3 via uL23, which was replaced in the rabbit network by uL14. Path number two conserved rProtein families uL5 and uS13. The connection in yeast was bridged via uL11. Path number three conserved two connective edges, eL30-uS15 and eL19-uS17. Finally, path number four conserved the connection between uS1, and nodes eL2 and eL8. The latter had lower weights between the interconnected edges. Each subunit had highly interconnected neighborhoods that formed around hubs, i.e., nodes with high degree and high interconnectivity among neighbors (high clustering coefficient). The conserved center of the LSU and node with largest degree was uL4. The PET stemmed right from uL4 and elongated to interface path number three. Next to uL4, nodes uL30 and eL20 acted as hubs to connect other highly interconnected neighbors from the LSU. Node eL20 connected the P-Stalk structure and its surrounding area, while uL30 connected both eL20 and uL4. The conserved centers of the SSU were uS3 and uS5. Both centers connected rProtein-condensed regions. Additionally, in the rabbit network, uS8 was the SSU node with the highest degree. Node uS3 positioned in the edge path stemming towards interface path number two while uS5 toward interface path number one and three. Overall, regions in the SSU were more compact and separated from each other as compared to regions in the LSU, which were evenly interconnected impairing visual separation.

As a verification step, we assessed the extent of defined regions and their match to biologically known ribosomal features outlined in the network topologies (Table 3), which gave us a clear understanding of the constraints and potential of our method during coherent regional definitions.

The defined regions (Fig. 5) reflected the overall structural organization of ribosomes. First, there were inter-subunit connective paths, which comprised interface regions defined on three highly weighted paths in yeast and rabbit (Table 3). There was a fourth path, characterized by eS1, which had a lower relative weight of the edges that connect subunits. Thus, the eS1 inter-subunit path was not defined as a region by our method. Within the subunit mainland, LSU in both test cases contained more regions than SSU, reflecting a higher edge number between nodes. Consequently, LSU regions contained more overlaps and less unique nodes. The SSU, on the other hand, formed communities, subsetting the SSU node-set into a less overlapped set of regions. In brief, defined LSU regions were structurally related to the PET, a central region, interface-adjacent regions and a subunit top-region. The latter positioned itself besides the P-Stalk. SSU regions divided into a central hub (i.e., uS3-containing region), a bottom tail that stemmed from the central hub, a central region that contained uS5 and interface adjacent regions. The center was defined as a separate region in the rabbit network but in the yeast network was attached to the interface regions.

Subsetting structures from multi-protein complexes into regions as performed by ${\text{COSNet}}_i$ is equivalent to detecting communities inside of a network. Therefore, we compared the performance of ${\text{COSNet}}_i$ to pick coherent regions or communities to that of publically available algorithms. Three types of algorithms were tested. One based also on random walks, i.e., walktrap, a second one based on the map equation, i.e., Infomap, and, in addition, a third one based on eigenvectors, i.e., eigenvector based models. The three tested algorithms (see tests details in Additional file 5 for walktrap and eigenvector models, and in *Region_selection_infomap.py* for Infomap) have a crucial conceptual difference with ${\text{COSNet}}_i$, which is that nodes may not be redundant within communities, with the consequence that community size varies considerably (Additional file 6). This feature may be more or less desirable depending upon the experimental or biological question. Walktrap finds 11 communities both in the rabbit and yeast networks, varying in size from two to 17 nodes. The eigenvector method finds eight communities both in the rabbit and yeast networks, varying in size from one to 24 nodes, and Infomap finds 11 and nine communities in the rabbit and yeast networks, respectively, varying in size from three to 17 nodes. In terms of network topology, the regions or communities match to those reported in Table 3 by conserving the same node kernels or region core, with the main difference being the number of nodes per region. As an example, the PET region (highlighted green in Additional file 6), characterized at least by nodes eL39 and eL37, varies considerably in size and composition with each algorithm even though it conserves the same node core. For rabbit, the PET regions are always consistent with ${\text{COSNet}}_i$ PET 1 and 2 but also reach beyond to borrow nodes from the adjacent central LSU regions. For yeast, the PET regions are pronouncedly variable in size and reach all over the LSU adjacent regions.

### Testing the spatial constraints of ribosome specialization

**Fig. 6:**
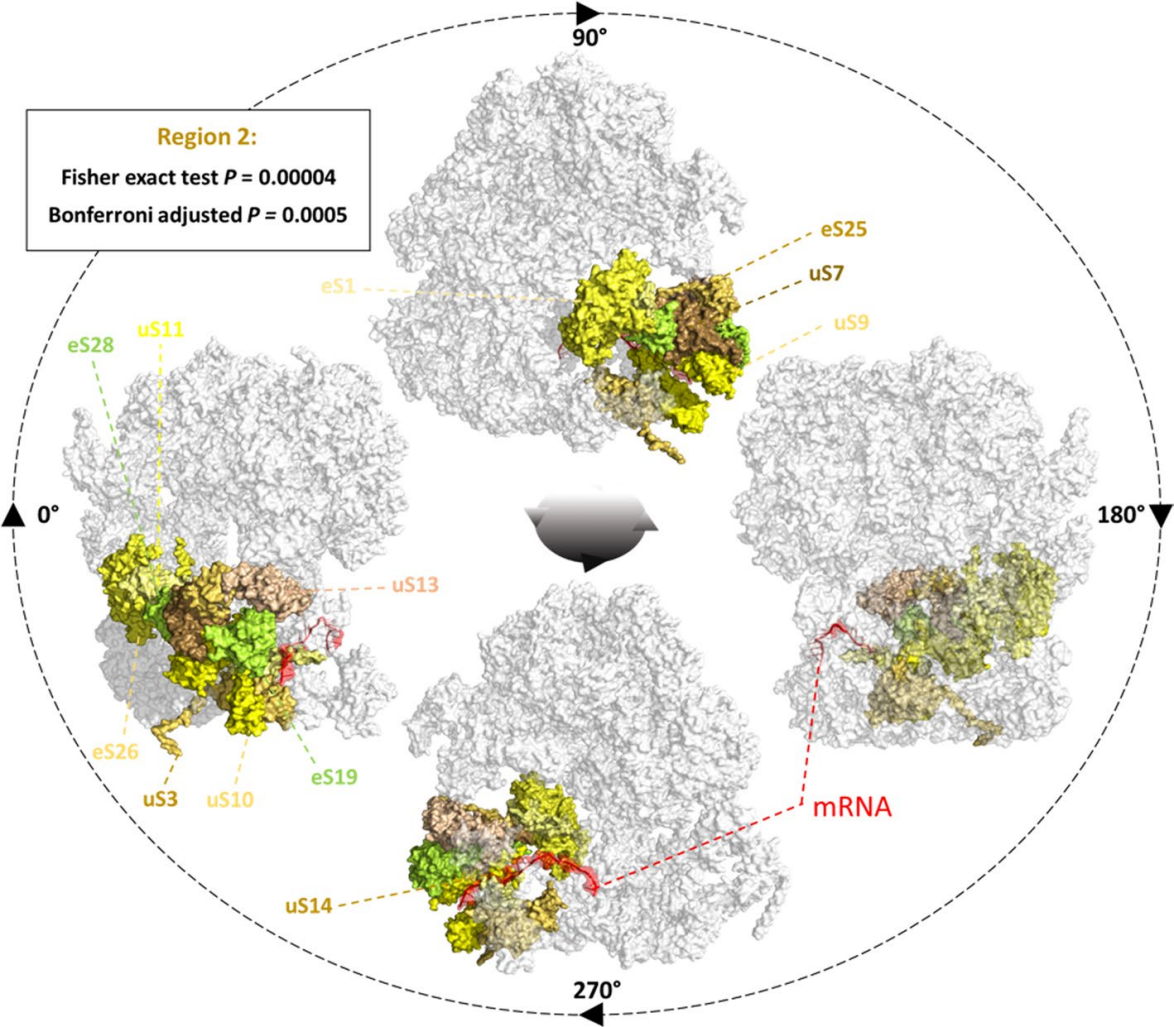
Spatial confinement during ribosome specialization: a test case of the COSNet$_i$ workflow. Optimized conditions were used to test whether the distribution of substoichiometric proteins is significantly constrained to specific ribosomal regions in yeast and mammalian systems. The weighed graph used to select regions was optimized as detailed in Fig. 5. The code commands used to produce our results are outlined in Code Chunk 1. The mammalian and yeast systems were tested and only the yeast depleted substoichiometric rProteins were significantly localized in a SSU region (colored in yellow shades) after Bonferroni correction of the Fisher exact test *p*-values (i.e., Region 2: P = 0.00004, Padj = 0.0005). The mRNA has been colored red to outline its relative location as compared to the region enriched in depleted proteins. Ribosomal structures are rotated 90$^{\circ }$ in the y-axis at a time in order to visualize the boundaries of the significantly changed region

Multi-protein complexes such as ribosomes can undergo changes in their associated structural rProteome. Variability from a canonical rProteome composition is known as substoichiometry. Deviations that qualify as substoichiometry can relate to subtractional heterogeneity, i.e., lost rProteins [43], also to exchanged rProtein paralogs [7, 18], to differential composition of immature and mature complexes [44], among others. In our test cases, rProtein substoichiometry has been linked to specialized ribosomal roles. Thus, we used the reported significantly substoichiometric rProteins as positive (“1”) binary input in our method while the rest of the ribosomal proteome was set to negative (“0”) or not changed.The percentage of total significantly changed rProteins was 22% and 15% for the mammalian and yeast systems, respectively (Additional file 4). In mammalian ribosomes, three subcategories could be created that comprise 8% (total substoichiometric rProteins), 4% (substoichiometric rProteins in non-translational ribosomal complexes) and 3% (substoichiometric rProteins in translational ribosomal complexes). With an 8% background significance, 13 rProteins per region are needed to test significances, implying a large number of steps in the walking length. This is already at the boundary of node proportion for test sampling. Going below 8% required a random walk of more than 50% of the nodes and thus neglected the capability of our method by picking up the entire subunits as coherent regions. In the yeast test case, two subcategories could be created that comprised 7% each of background significance ([1] significantly enriched and [2] depleted). Seven percent of background significance needs defined regions of 14 rProteins in average and thus the node proportion is still acceptable. Thus, the prioritized tests (see Code Chunk 1), two for mammalian and three for yeast, avoided those that had a low background significance in the mammalian system. The test specifics: region average size (RAS), background significance (BS), walking lengths (WL) and threshold ($d_t$) are compiled in Code Chunk 1. The binary columns used to run the *intcryomics.py* function are reported in Additional file 4. Statistically relevant results from testing the binary files on the optimized rabbit and yeast networks (see Building a Ribosomal Protein Network) are outlined in Fig. 6.

#### Code chunk 1

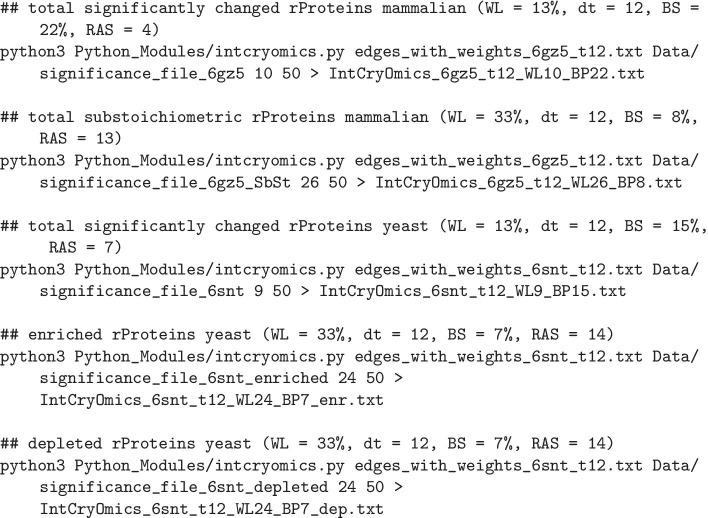



We found spatially constrained rProtein substoichiometry in the yeast network. More specifically, in the subcategory of depleted substoichiometric rProteins. Our results support the notion that depleted rProteins from actively translating polysomes as compared to monosomes, in glucose fed yeast growing at stationary rate, are significantly constrained to the 40S SSU region adjacent to the mRNA and tRNA entry points. The structural coherence of the resulting region is evident when multiple graph community detection algorithms are used in the yeast rProtein network (highlighted in yellow in Additional file 6). Infomap-, walktrap-, and eigenvector-based models all yielded the same 40S SSU region, namely consisting of nodes uS3, uS14, eS10, eS17, and uS10. The difference with the ${\text{COSNet}}_i$-derived regions is the lack of some significantly changed nodes, such as eS1, in the alternative algorithms, which further emphasizes the importance of ${\text{COSNet}}_i$’s flexibility during region definition.

Two more regions exhibited significant *p*-values, but were no longer significant after the stringent Bonferroni correction. The regions both belonged to the yeast test case, one at the total substoichiometric category, and the other at the enriched substoichiometric category. The regions were overlapping heavily, the total constituent rProteins were: [‘eL31’, ‘uL22’, ‘eL6’, ‘eL20’, ‘eL33’, ‘uL14’, ‘uL16’, ‘uL6’, ‘eL32’, ‘eL40’], The *p*-values went from 0.025 and 0.016 to 0.477 and 0.177 after Bonferroni correction. In the mammalian system, no significant *p*-values were obtained.

## Discussion

${\text{COSNet}}_i$ contributes to the field of structural systems biology [20], where structural and system biology approaches converge to contextualize shifts in omics abundances from molecular species that belong to a multi-protein complex. ${\text{COSNet}}_i$ translates multi-protein complex atomic structures into weighted graphs. Typically, these type of network analyses have been used to capture inter-residue interactions and provide structure to function insights in individual protein structures [21] [45–47]. Here, we extend the approach to study interactions between proteins that belong to multi-protein complexes. In order to avoid prior-knowledge biases during region selection, the defined interactions are a proximity probability and not inferred based on domain knowledge of the proteins that comprise the studied complex. A random walk with restart methodology is used to define structurally coherent regions as opposed to regions of biological interest defined based on known domains and accessory proteins (Example of the latter in Woolford et al. [3]). Regarding the integration to systems biology omics data, we propose a structural contextualization that goes beyond estimating significances of protein abundance changes. An initial mapping of protein changes onto structures of complexes can already indicate whether the changed components are spatially adjacent or may have any obvious functional implications. Our goal was to devise a methodology that enables asking whether relevant protein changes are significantly confined to specific parts of a complex. The proposed approach is built under the null hypothesis that the proportion of changes between the whole complex and randomly selected regions are not different. In other words, that the significantly changed molecular species are randomly scattered across the structure. To test deviations from the background proportion of significances, ${\text{COSNet}}_i$ uses the Fisher exact test [48, 49]. This test allows for the significance value of mean deviations to be calculated exactly.

### Structure quality requirements

**Fig. 7:**
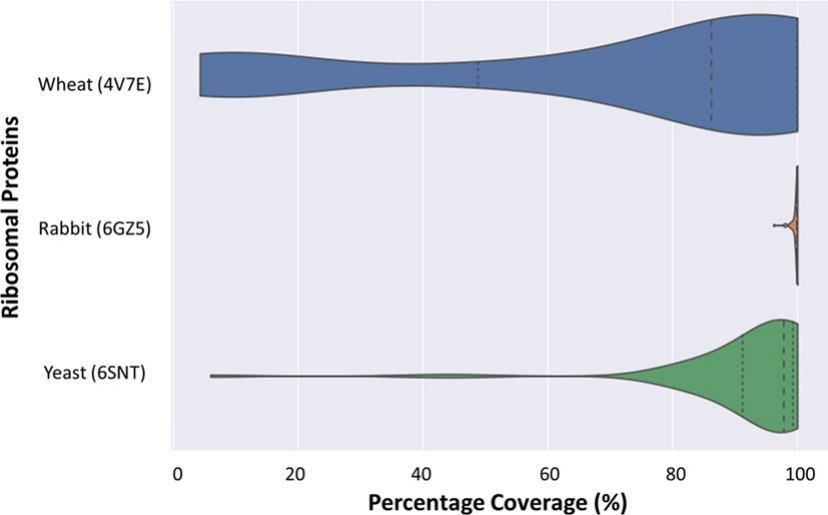
Violin plots of rProtein sequence percentage coverage in interpreted Cryo-EM densities of cytosolic ribosomes. Structures are derived from PDBx/mmCIF entries 6SNT, 6GZ5 and 4V7E corresponding to *Saccharomyces cerevisiae* (bottom), *Oryctolagus cuniculus* (middle) and *Triticum aestivum* (upper) ribosome structures. The percentage of coverage per rProtein was calculated using the *check_cif_completeness.py* from the ${\text{COSNet}}_i$ methodology

${\text{COSNet}}_i$ starts its procedure from an experimentally elucidated complex structure. The structure needs to fulfill quality parameters, especially those regarding accurate placement of the protein features. Accurate representation of the protein positions within a multi-protein complex is influenced by the cryogenic or crystallographic resolution. Atomic models can be effectively built at resolutions below to 4 Å [50–53]. Additionally, at low resolutions the models tend to overfit the data. This is a recognized problem that has been addressed in multiple ways [54]. The implication for mmCIF/PDBx files is that, when overfitted, there might not be sufficient sequence coverage for some of the proteins that are actually modelled onto the structure. In order to evaluate this, we provide users the quality assessing script *check_cif_completeness.py*. The script compares the coverage of the modeled sequences relative to the original FASTA sequence of the protein. As a working example of the ribosomal test case, the *Triticum aestivum* 80S structure can be taken, which is relatively poorly resolved as compared to the yeast and rabbit counterparts. A threshold of 12 Å does not achieve connectivity of the P-Stalk feature, while outlier proteins already appear in the defined regions (Fig. 3). Thus, a good consensus between connectivity and lack of outlier proteins or island regions seems unlikely for the wheat ribosome structure. Additionally, when the model was interrogated by *check_cif_completeness.py*, it became clear that many rProteins have a low sequence coverage in this structure (Additional file 7A and Fig. 7).

We recommend users inputting into the method the best available resolved structure for the investigated complex. Accurate protein densities will translate into a reliable weighted graph that allows structure-directed region selection. Additionally, we recommend that users make use of the *check_cif_completeness.py* in order to assess the general quality of the protein features modelled onto the initial structure densities. The preferred outcome would be modeled protein sequences that fully overlap with the FASTA sequences that are reported for those proteins, just as what we report here for the yeast and rabbit modelled rProteins (Additional file 7B and C).

### Optimization of region definition

The random walk procedure used by ${\text{COSNet}}_i$ belongs to the most standard types of stochastic walks, i.e., moving through a network with a probability that equals the edge weights [55]. ${\text{COSNet}}_i$ subsets the original graph and tests properties of the subset-nodes as compared to the node set as a whole. Thus, the region selection procedure must not violate the independence assumption implicit to the proportion test [56]. The assumption is not violated because networks are based on spatial distances and not similarities or dissimilarities between empirically measured omics data. The latter produces indirect protein interaction networks that outline relationships of shared functionality and interdependence [57]. Thus, ${\text{COSNet}}_i$ samples node clusters independently without any bias from shared functionality.

Defined regions are based on an adjacency matrix of protein-protein interactions calculated at a distance threshold of predefined Ångströms (Å). The distance threshold can be selected based on the quality of the resulting regions, e.g., connectivity, biological accuracy, lack of outlier node-components. Thus, a quality metric to assess the quality of defined regions is their resemblance to characterized features of the investigated complex. Additionally, users can rely on the established consensus distance of 8Å between amino acid residues within a protein structure [32] or, equally legitimate, on elucidated contacts from empirical evidence. The structure-translated graph features edges that are weighted by the percentage of contact coverage among nodes indicating a transit probability, which probabilistically guides the random-walk path trajectory. Edge weights are the single most influencing attribute in the outcome of a random walk through a weighted graph [58] and as such are the attribute that enables structural coherence in our method. This property of the edges bounds the region-definition process to the network topology [59], which ultimately depends in the original structure.

Other parameters influencing the region definition process are the walking length and the iteration number. Generally, increasing the iteration number achieves region consensus in the defined regions (Fig. 4). Regarding the walk length, in our test case we propose optimizing it according to the omics data to be tested. More specifically, since the walking length affects the region size, we aimed at an average region size that allowed for a single feature within a selected region to equal in proportion the baseline proportion of significances. In this way, we avoided overweighting a single significance beyond its actual importance. In practice, going beyond the test case provided will allow users to vary the walk length for different inquiries. For instance, increasing the walking length implies uncovering structural features of the network [55, 60] that identify central nodes, edges or other community substructures [61] in the original network. Thus, our algorithm can be used without any omics tests in order to investigate the very nature of node communities at different scales within the entire graph.

Graph community detection algorithms [62, 63], as those used here to compare to ${\text{COSNet}}_i$, are suited for the detection of non-overlapping coherent regions. These regions or communities can be used to validate observations made with ${\text{COSNet}}_i$ about the network topology. This validation in turn reveals robust topological aspects of the biological networks under study. At the same time, due to the intrinsic nature of the alternative algorithms, the selected communities tend to be largely of different sizes, so that a quasi-standardized number of nodes is unlikely to be obtained. This feature precludes their further use with ${\text{COSNet}}_i$ if the background significances require a specific region size. Nevertheless, there are many experimental scenarios that are not limited by background significance. In these cases, the non-overlapping set of regions can even be used to follow up the ${\text{COSNet}}_i$ algorithm. The synergy of the innovations implemented by ${\text{COSNet}}_i$ and the capabilities of the existing graph community detection algorithms creates a comprehensive set of tools for studying decomposed networks from multi-protein complexes.

In our test case, it is necessary to consider the biology of ribosome specialization, in which different sub-populations of rProtein-enriched ribosomes can selectively translate transcripts [7, 10]. In this context, approximately equally-sized ribosomal regions with the right combinations of rProteins might be more relevant than analysis of strictly non-overlapping regions of highly diverse size to study the phenomenon of specialization. Therefore, continuing the ${\text{COSNet}}_i$ workflow with a minimal set of overlapping regions as defined in our method would be the first choice. Additional information on ribosomal protein networks and their internal community topology may then be inferred from the comparison to regions obtained by existing graph community detection algorithms.

### Ribosomal networks

RNA physically mediates many contacts in the interaction network of ribosomes [23]. Thus, the 8 Å consensus, a catch-all type of threshold under which Van der Waals, hydrogen bonding, electrostatic interactions can occur, can be increased to include those rRNA-mediated contacts as edges. A threshold of 12 Å achieves more than 95% of interconnected nodes in the yeast and rabbit networks while avoiding outlier proteins. Regarding the random walk parameters: (1) an iteration number of 50 consensus samplings avoided bias towards outlier rProteins. (2) A customized walking length to the conducted tests allowed increasing the step number until covering 33% of the node set in a single walk. The ribosomal networks represented as weighted graphs resemble the topology and relative distribution of rProteins in the actual 3D structure. This becomes clearer when the layout is deterministically defined by minimizing the weight (edge) total force on the networks (Fig. 5) according to the Kamada-Kawai Algorithm [64] as applied in Cytoscape [65]. Thus, the selected thresholds become further validated.

**Fig. 8:**
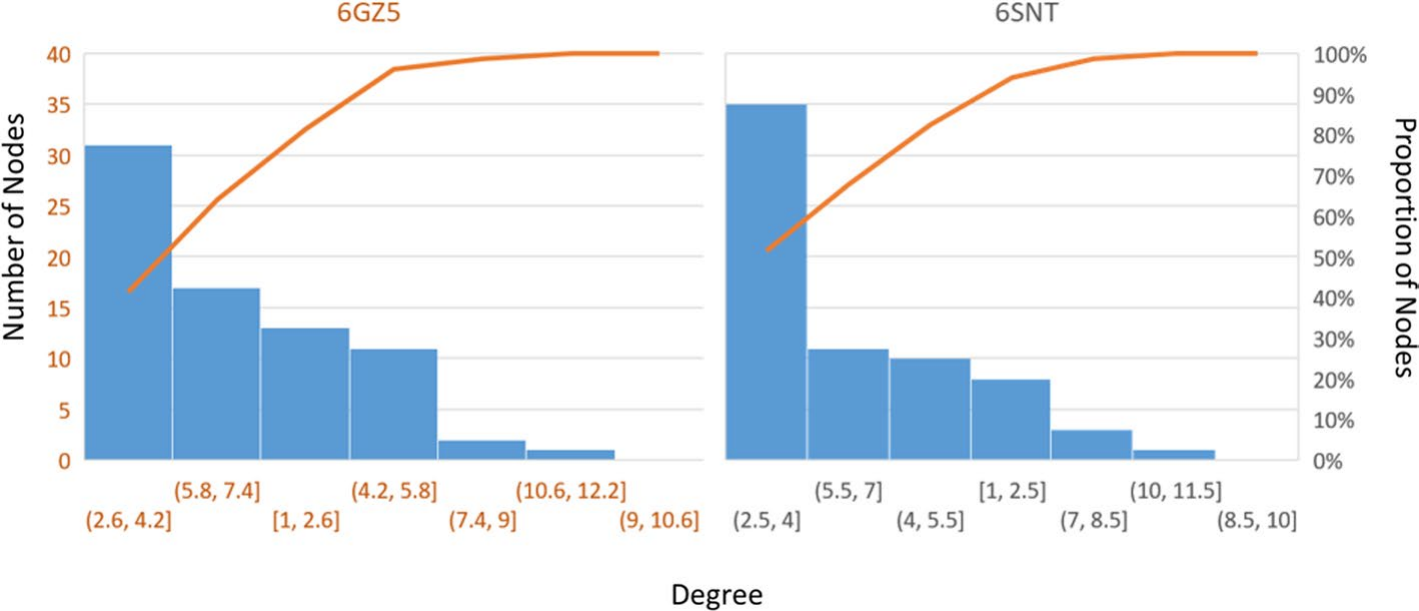
Histograms summarizing the node degree statistics of the optimized rabbit and yeast ribosomal protein networks. Networks were analyzed in Cytoscape [34]. Distributions of node degrees were plotted in Pareto-scaled histograms featuring the number of nodes on the left y-axis and the proportion of nodes on the right y-axis. Note that in both cases a heavy-tailed distribution peaking at a range of 2.5–4.5 degree characterizes more than 30% of the nodes in both networks. As in other figures and supplemental tables, the rabbit network (6GZ5) is identified by red font and the yeast network (6SNT) is identified by black font

In the two tested ribosomal networks, degree distributions are heavy-tailed and almost identical (Fig. 8). “Heavy-tailed” means that density histograms from degree distributions will reach zero later than expected by an exponential function [66]. This implies that several nodes with high degrees dominate the tail of the distribution histograms. Upon inspection, these nodes are hubs in densely packed graph subsets, and as such, could influence their surroundings rather than be affected individually by any regulatory mechanism. Hub removal could cause major disruptions to the structural stability of ribosomes, which can be aggravated by the propensity of rProteins to aggregate [67]. Promiscuous binding into aggregates occurs due to the rProtein own basic nature that enhances rRNA binding [68, 69].

The tested ribosomal networks also share interconnected paths through similar edges, i.e., influential hubs that connect modules or communities. Thus, the topology seems to be well conserved between yeast and rabbit cytosolic rProtein networks. From an evolutionary perspective, both networks should be conserved since the main rProteome acquisitions are shared [12]. An exception is that higher metazoans (except plants) share an increase of basic LSU rProtein components as compared to lower eukaryotes [14]. Importantly, in such a conserved system, ${\text{COSNet}}_i$ finds equivalent regions when the parameters are tuned equally. The resulting regions contain shared rProteins, especially in the highly interconnected neighborhoods. Overlaps imply that significantly changed regions after Bonferroni correction of the initial Fisher test *p*-values do not represent a fixed, isolated region. Rather, significance might be tied to the right combination of interconnected rProteins, which could be targeted by non-random complex remodeling or de novo synthesis of components.

### Spatially enriched ribosomal protein substoichiometry

Using the ${\text{COSNet}}_i$ workflow, we tested previous claims of rProtein-dependent ribosome specialization in mammalian [37] and yeast [39] systems. We aimed at uncovering if the specialized complexes feature spatially enriched regions in substoichiometric proteins. We found that significantly depleted substoichiometric rProteins in yeast polysomes are spatially constrained. The interrogated polysomes correspond to four monosomes loaded into an mRNA. Thus, the substoichiometric complexes were translationally competent, and as such, an altered ribosomal region in those complexes might signal functional features that feedback on translation. Interestingly, substoichiometric rProteins were constrained to a region in the 40S SSU that lies at the tRNA exit and entry sites and is adjacent to the mRNA entry channel. This observation increases the possibility of the regulation found being a targeted response to modify translational preferences toward certain transcripts. Moreover, the same region seems to be depleted in rProteins, according to quantitative structural analyses, in yeast shifted from glucose to glycerol medium [70]. Ribosomes with depleted proteins were already visible in glucose-fed yeast, but increased when shifted to glycerol. The previous observation was made in enriched ribosomal pellets with contributions from polysomes and monosomes. Conversely, the exemplary yeast dataset presented here accounts for substoichiometry of heavily loaded polysomes as compared to monosomes. Thus, the most likely linking explanation is that a balance between rProtein-depleted translationally competent complexes and monosomes that are not depleted from the mentioned rProteins, is necessary upon shifting yeast from glucose to glycerol as a carbon source. In order to evaluate whether the findings outlined in Sun et al. [70] are significantly constrained to the same region outlined in Fig. 6, we deliver a variation of the *intcryomics.py* function named *intcryomics_sigassign.py*, the function only needs as input an edge list, a walking length and an iteration number. The output are defined regions, which the user can then select and input which proteins are significantly changed. Finally, the Fisher test is performed, *p*-values are adjusted and the function returns as output a logical string accompanied by Bonferroni *p*-adjusted values indicating whether the input proteins are significantly constrained to the selected region. The test determined that rProteins uL16(RPL10), eS1(RPS1), uS11(RPS14A/B)) and eS26(RPS26A/B), lacking in 80S ribosomes as detailed in Sun et al. [70], are significantly constrained to the same region outlined in Fig. 6 with a Bonferroni *p*-adjusted value always below 0.005.

Different possibilities could explain significantly depleted rProteins as a functional mechanism to modulate translation: (1) rProteins that cannot be associated with actively translating ribosomes because they imply translational restrictions for some transcripts. Functional depletion of rProteins has been described as subtractional heterogeneity [43]. (2) Alternatively, rProteins could be tightly bound to other ribosome associated factors that assist mRNA recruiting (e.g., [71]). Tight interactions could cause force on rProtein links during ribosome purification, and rProteins could then be systemically lost from polysomes. (3) Another alternative is that rProteins have extra-ribosomal functions directly or indirectly involved in mRNA recruiting (e.g., [72, 73]), for which they could be depleted from polysomes while involved in the formation of initiation complexes.

## Conclusion

${\text{COSNet}}_i$ achieves a structure-directed partitioning into regions within any multi-protein complex for which a sufficiently resolved structure exists. A plant cytosolic ribosome structure is needed to increase the quality of the current rProtein network without compromising isolated regions. By contrast, the yeast and rabbit ribosomal networks could be successfully used to optimize the ${\text{COSNet}}_i$ parameters. Optimization of distance threshold to call proteins to be in contact, walking length to define regions and consensus sampling iterations largely depend on the type of multi-protein complex investigated and the structure resolution. The optimization makes use of prior knowledge of the investigated complexes and is influenced by the observed number of significant abundance changes. Finally, using the ${\text{COSNet}}_i$ fully optimized method, we scrutinized previous claims of specialized ribosomes. More specifically, we tested whether the rProtein-dependent claims could be traced to a specific ribosomal region being modulated. For this purpose, we used the minimal set of overlapping regions covering most rProtein nodes, as inferred by ${\text{COSNet}}_i$, instead of using non-overlapping different sized regions, as determined by existing graph community detection algorithms. The latter set of regions or communities allowed us to validate the topology of the ribosomal protein networks. We found that indeed subtractional heterogeneity is confined to the tRNA exit and entry sites in actively translating yeast polysomes. Furthermore, based on validation by three independent graph community detection algorithms, we conclude that the regulated region is structurally coherent. Thus, the constraint might signal functional features of translation, i.e., depleted spatially related structural rProteins influencing the translational status of transcripts in yeast fed with different carbon sources. Our method has been made publicly available as a GitHub repository (https://github.com/MSeidelFed/COSNet_i) and can be installed using the python package installer pip.

## Availability and requirements

- Project name: ${\text{COSNet}}_i$—ComplexOme-Structural Network Interpreter
- Project home page: https://github.com/MSeidelFed/COSNet_i
- Operating system(s): Platform independent
- Programming language: Python
- Other requirements: Python-3.6.5 or higher, numpy 1.18.1 or higher, biopython 1.78 or higher, os-sys 2.1.4 or higher, scipy 1.5.2 or higher, networkx 2.5 or higher, matplotlib 3.3.1 or higher.
- License: BSD 2-Clause “Simplified” License.
- Any restrictions to use by non-academics: None.

## Supplementary information

**Additional file 1**. COSNet_i_ python module USAGE.md file for the integration of relative changes obtained by omics-technologies into Cryo-EM or crystallography based randomly sampled interaction networks of multi-protein complex structures. The module is composed of independent components, written as python scripts (found in the Modules folder), which can be run in batch with bash or python scripts (bash scripts are found in the Batch files’ folder).

**Additional file 2**. Nodes and edges structure of ribosomal networks from mmCIF entries 6SNT, 6GZ5 and 4V7E corresponding to *Saccharomyces cerevisiae* (Column N-S), *Oryctolagus cuniculus* (Column H-M) and *Triticum aestivum* (Column B-G). Each cell corresponds to one string representing a single contact that can be separated by the space character. The first element of the string is the source node, the second element the target node, the third element the number of contacts between them (edge weights), the fourth and fifth columns are color identifiers for the nodes in the networks as outlined in Figure 3.

**Additional file 3**. Defined ribosomal regions at different walking lengths (WL). Optimized parameters were used, the °Angström threshold was 12, and the iteration number of the consensus sampling was 50 iterations. Tab A contains regions derived from the 6SNT yeast ribosome structure. Tab B contains regions derived from the 6GC5 rabbit ribosome structure. Both tabs contain the selected regions at varying walking lengths, from which Table 2 was built.

**Additional file 4**. Baseline proportions for statistical testing of spatial enrichment in ribosome multiprotein complexes test case. (Columns F-H) Mammalian binary input necessary for intcryomics.py featuring changed rProtein paralogs with 1 and non-changed with 0. (Columns K-N) Yeast binary input necessary for intcryomics.py featuring changed rProtein paralogs with 1 and non-changed with 0. Non-tested cases are signaled with grey font. Prioritized and tested case are signaled with black font.

**Additional file 5**. Community Detection within Graphs. R implementation of walktrap and eigenvector based models algorithms.

**Additional file 6**. Modules or communities found in optimized ribosomal networks from mmCIF entries 6SNT and 6GZ5 corresponding to *Saccharomyces cerevisiae* (Column H-M) and *Oryctolagus cuniculus* (Column B-G). Three different contrasting algorithms were used to find communities across optimized ribosomal protein graphs. Namely, Infomap, walktrap and eigenvector based models. The resulting regions were then used to compare to those obtained from the COSNet_i_ procedure as outlined in Table 3. Green highlights the exemplary PET region as was picked out by each algorithm.

**Additional file 7**. Percentage of sequence coverage modelled into the exemplary mmCIF/PDBx structures used to optimize the COSNet_i_ workflow. (A) The yeast (*Saccharomyces cerevisiae* PDB ID: 6snt), (B) rabbit (*Oryctolagus cuniculus* PDB ID: 6GZ5) and (C) plant (*Triticum aestivum* PDB ID: 4v7e) ribosomal complexes. The assessment can be replicated in any structure using the python function check_cif_completeness.py as documented in (https://github.com/MSeidelFed/COSNet_i).

## Data Availability

The datasets generated and/or analysed during the current study are available in the ${\text{COSNet}}_i$ repository, https://github.com/MSeidelFed/COSNet_i/tree/master/Data. Additionally, the datasets supporting the conclusions of this article are included within the article (and its additional files).

## Abbreviations

${\text{COSNet}}_i$: ComplexOme-Structural Network Interpreter
rProteome: Ribosomal Structural Proteome
rProtein: Ribosomal Protein
ES: Expansion Segments
RAS: Region Average Size
PET: Polypeptide Exit Tunnel
BS: Background Significance
WL: Walking Lengths
$$\hbox {d}_t$$: Distance Threshold
Å: Ångströms
SSU: Small Subunit
LSU: Large Subunit
CRA: Changed in Relative Abundance
