# Supplementary material for: COSNeti: ComplexOme-Structural Network Interpreter used to study spatial enrichment in metazoan ribosomes
Source: BMC Bioinformatics. 2021 Dec 20;22:605. doi: 10.1186/s12859-021-04510-z (PMC8686616; doi:10.1186/s12859-021-04510-z)
Supplement: Supplementary file 5 — Additional file 5. Community Detection within Graphs. R implementation of walktrap and eigenvector based models algorithms. [file 12859_2021_4510_MOESM5_ESM.html]

Community Detection within Graphs


# Community Detection within Graphs

#### Federico Martinez-Seidel, Yin-Chen Hsieh

#### 05 11 2021

## igraph package and other dependencies / functions needed

```
list2df <- function(x) 
{ 
   MAX.LEN <- max(sapply(x, length), na.rm = TRUE) 
   DF <- data.frame(lapply(x, function(x) c(x, rep(NA, MAX.LEN - length(x))))) 
   colnames(DF) <- paste("V", seq(ncol(DF)), sep = "")   
   DF 
} 

library(reshape2)
```

```
## Warning: package 'reshape2' was built under R version 3.6.3
```

```
### installation

#### install.packages("igraph_1.2.7.zip", repos = NULL, type = "win.binary")

library('igraph')
```

```
## Warning: package 'igraph' was built under R version 4.2.0
```

```
## 
## Attaching package: 'igraph'
```

```
## The following objects are masked from 'package:stats':
## 
##     decompose, spectrum
```

```
## The following object is masked from 'package:base':
## 
##     union
```

## 6gz5

```
Full_Network_6gz5 <- read.delim("edges_with_weights_6gz5_t12.txt",
                                sep = " ")

weights_6gz5 <- as.numeric(Full_Network_6gz5[,3])

graph_6gz5 <- graph_from_edgelist(el = as.matrix(Full_Network_6gz5[,1:2]),
                                  directed = FALSE)
```

### Walktrap

```
test_WT_6gz5 <- cluster_walktrap(graph = graph_6gz5,
                                 weights = weights_6gz5,
                                 steps = 10)

#### modularity (range between -1 and 1;
             ## higher values indicate division of a graph into modules)

modularity(test_WT_6gz5)
```

```
## [1] 0.7374091
```

```
#### groups

groups(test_WT_6gz5)
```

```
## $`1`
##  [1] "eL13" "eL18" "eL36" "uL15" "uL1"  "uL29" "uL4"  "eL15" "eL8"  "eL28"
## [11] "eL32" "eL31" "uL22" "eL39" "eL37" "uL23" "uL24"
## 
## $`2`
##  [1] "eL14" "eL20" "eL6"  "uL13" "uL6"  "eL21" "eL29" "uL30" "eL33" "uL16"
## [11] "uL18" "eL40"
## 
## $`3`
## [1] "eS1"  "uS9"  "eS26" "uS11" "eS25" "uS7"  "eS28"
## 
## $`4`
## [1] "eL19" "eL22" "uS17" "uS4"  "eS24" "eS4"  "eS30" "uS12" "eS8" 
## 
## $`5`
## [1] "eS7"  "uS15" "eS17" "uS2"  "eS21" "eS27" "uS5"  "uS8" 
## 
## $`6`
## [1] "eL42" "uL5"  "eS19" "uS13" "uS19"
## 
## $`7`
## [1] "uL2"  "eL34" "eL27" "eL30" "eL43"
## 
## $`8`
## [1] "eL24" "eS6"  "uL14" "uL3" 
## 
## $`9`
## [1] "uL10" "uL11"
## 
## $`10`
## [1] "eS10" "uS14" "uS3"  "uS10"
## 
## $`11`
## [1] "eS12" "eS31"
```

```
write.table(na.omit(melt(t(list2df(groups(test_WT_6gz5))))),
            "test_WT_6gz5.txt", sep = "\t", dec = ".")

#### dendrogram

plot_dendrogram(test_WT_6gz5, cex = 0.3)
```

### Eigenvector

```
test_EV_6gz5 <- leading.eigenvector.community(graph = graph_6gz5,
                                              weights = weights_6gz5,
                                              steps = 10)

#### modularity (range between -1 and 1;
             ## higher values indicate division of a graph into modules)

modularity(test_EV_6gz5)
```

```
## [1] 0.6969078
```

```
#### groups

groups(test_EV_6gz5)
```

```
## $`1`
##  [1] "eL13" "eL18" "eL36" "uL15" "uL1"  "uL29" "uL4"  "eL15" "eL42" "eL28"
## [11] "eL32" "uL22" "eL39" "eL37" "uL23" "uL24"
## 
## $`2`
##  [1] "eS7"  "uS15" "uS17" "eS10" "uS14" "uS3"  "eS17" "uS2"  "eS21" "eS27"
## [11] "uS4"  "uS5"  "uS8"  "eS24" "eS4"  "eS30" "uS12" "uS10"
## 
## $`3`
## [1] "eL8"  "uL2"  "eL34" "eL27" "eL30" "eL43"
## 
## $`4`
##  [1] "eL14" "eL20" "eL6"  "uL13" "uL6"  "eL21" "eL29" "uL30" "eL33" "uL16"
## [11] "uL18" "eL40"
## 
## $`5`
## [1] "eL22" "eL31" "uL10" "eS12" "eS31" "eS8"  "uL11"
## 
## $`6`
## [1] "eL19"
## 
## $`7`
##  [1] "uL5"  "eS1"  "eS19" "uS13" "uS9"  "eS26" "uS11" "eS25" "uS7"  "eS28"
## [11] "uS19"
## 
## $`8`
## [1] "eL24" "eS6"  "uL14" "uL3"
```

```
write.table(na.omit(melt(t(list2df(groups(test_EV_6gz5))))),
            "test_EV_6gz5.txt", sep = "\t", dec = ".")

#### dendrogram

plot_dendrogram(test_EV_6gz5, cex = 0.3)
```

## 6snt

```
Full_Network_6snt <- read.delim("edges_with_weights_6snt_t12.txt",
                                sep = " ")

weights_6snt <- as.numeric(Full_Network_6snt[,3])

graph_6snt <- graph_from_edgelist(el = as.matrix(Full_Network_6snt[,1:2]),
                                  directed = FALSE)
```

### Walktrap

```
test_WT_6snt <- cluster_walktrap(graph = graph_6snt,
                                 weights = weights_6snt,
                                 steps = 10)

### modularity (range between -1 and 1;
             ## higher values indicate division of a graph into modules)

modularity(test_WT_6snt)
```

```
## [1] 0.7483418
```

```
### groups

groups(test_WT_6snt)
```

```
## $`1`
##  [1] "eL21" "uL10" "uL30" "uL4"  "eL29" "uL5"  "eL42" "uL18" "uL24" "uL11"
## 
## $`2`
## [1] "eL19" "uS17" "uS4"  "eS24" "eS4"  "eS30" "uS12" "eS8" 
## 
## $`3`
##  [1] "eL20" "eL33" "uL14" "uL16" "uL6"  "eL32" "eL31" "uL22" "eL6"  "eL40"
## 
## $`4`
## [1] "eL27" "eL30" "eL34" "uL2"  "eL43"
## 
## $`5`
## [1] "eS19" "uS13" "uS7"  "uS9"  "eS25" "eS28" "uS8" 
## 
## $`6`
## [1] "eL8"  "eL28" "eL36" "uL13" "uL15" "eL39" "eL37" "uL29"
## 
## $`7`
## [1] "eS7"  "uS15" "eS21" "eS27" "uS2"  "uS5" 
## 
## $`8`
## [1] "eL24" "eS6"  "uL23" "uL3" 
## 
## $`9`
## [1] "eS10" "uS14" "uS3"  "eS17" "uS10"
## 
## $`10`
## [1] "eS12" "eS31"
## 
## $`11`
## [1] "eS1"  "eS26" "uS11"
```

```
write.table(na.omit(melt(t(list2df(groups(test_WT_6snt))))),
            "test_WT_6snt.txt", sep = "\t", dec = ".")

### dendrogram

plot_dendrogram(test_WT_6snt, cex = 0.3)
```

### Eigenvector

```
test_EV_6snt <- leading.eigenvector.community(graph = graph_6snt,
                                              weights = weights_6snt,
                                              steps = 10)

#### modularity (range between -1 and 1;
             ## higher values indicate division of a graph into modules)

modularity(test_EV_6snt)
```

```
## [1] 0.6602576
```

```
#### groups

groups(test_EV_6snt)
```

```
## $`1`
##  [1] "eL19" "uS17" "eS21" "uS2"  "uS4"  "uS5"  "eS24" "eS30" "uS12" "eS8" 
## 
## $`2`
##  [1] "eL20" "eL21" "eL33" "uL10" "uL14" "uL16" "uL30" "uL4"  "uL6"  "eL29"
## [11] "uL5"  "eL8"  "eL28" "eL32" "eL36" "eL42" "uL13" "uL15" "uL18" "eL6" 
## [21] "eL37" "uL29" "uL24" "eL40"
## 
## $`3`
## [1] "eS7"  "eL27" "eL30" "eL34" "uL2"  "eL43" "uS15" "eL39" "eS27"
## 
## $`4`
##  [1] "uL11" "eS1"  "eS19" "uS13" "uS7"  "uS9"  "eS26" "uS11" "eS25" "eS28"
## [11] "uS8" 
## 
## $`5`
## [1] "eL24" "eS6"  "uL23" "uL3"  "eS4" 
## 
## $`6`
## [1] "eL31" "uL22"
## 
## $`7`
## [1] "eS10" "uS14" "uS3"  "eS17" "uS10"
## 
## $`8`
## [1] "eS12" "eS31"
```

```
write.table(na.omit(melt(t(list2df(groups(test_EV_6snt))))),
            "test_EV_6snt.txt", sep = "\t", dec = ".")

#### dendrogram

plot_dendrogram(test_EV_6snt, cex = 0.3)
```
